# Enhancement of gasworks groundwater remediation by coupling a bio-electrochemical and activated carbon system

**DOI:** 10.1007/s11356-019-04297-w

**Published:** 2019-02-09

**Authors:** Panagiotis Kirmizakis, Rory Doherty, Carlos A. Mendonça, Ricardo Costeira, Chris C. R. Allen, Ulrich S. Ofterdinger, Leonid Kulakov

**Affiliations:** 1 School of the Natural and Built Environment, Queen’s University Belfast, Stranmillis Road, Belfast, BT9 5AG UK; 2 Department of Geophysics, University of São Paulo, Rua do Matão, São Paulo, 1226 Brazil; 3 School of Biological Sciences, Queen’s University Belfast, Lisburn Road, Belfast, BT9 7BL UK; 4 Institute for Global Food Security, Queen’s University Belfast, Lisburn Road, Belfast, BT9 7BL UK

**Keywords:** Bio-electrochemical system (BES), Granular activated carbon (GAC), Gasworks-contaminated groundwater

## Abstract

Here, we show the electrical response, bacterial community, and remediation of hydrocarbon-contaminated groundwater from a gasworks site using a graphite-chambered bio-electrochemical system (BES) that utilizes granular activated carbon (GAC) as both sorption agent and high surface area anode. Our innovative concept is the design of a graphite electrode chamber system rather than a classic non-conductive BES chamber coupled with GAC as part of the BES. The GAC BES is a good candidate as a sustainable remediation technology that provides improved degradation over GAC, and near real-time observation of associated electrical output. The BES chambers were effectively colonized by the bacterial communities from the contaminated groundwater. Principal coordinate analysis (PCoA) of UniFrac Observed Taxonomic Units shows distinct grouping of microbial types that are associated with the presence of GAC, and grouping of microbial types associated with electroactivity. Bacterial community analysis showed that β-proteobacteria (particularly the PAH-degrading *Pseudomonadaceae*) dominate all the samples. *Rhodocyclaceae*- and *Comamonadaceae*-related OTU were observed to increase in BES cells. The GAC BES (99% removal) outperformed the control graphite GAC chamber, as well as a graphite BES and a control chamber both filled with glass beads.

**Electronic supplementary material:**

The online version of this article (10.1007/s11356-019-04297-w) contains supplementary material, which is available to authorized users.

## Introduction

Effective management of groundwater contamination requires the development of sustainable remediation technologies (Arias Espana et al. 2018; Ellis and Hadley 2009; Nathanail et al. 2017). These are technologies that consider environmental, social, and economic criteria such as cost effectiveness, technically proficiency, and social acceptance. The modification and optimization of socially accepted remediation technologies, such as sorption media into newer technologies that enhance and monitor degradation in near real time, make them ideal candidates as sustainable remediation technologies. Standard groundwater remediation approaches often focus on a specific area or system where the remediation effort is focused. In ex situ systems, this can be in the form of an engineered container or chamber that contains the method of remediation, such as sorption using GAC (Guerin 2008), other porous media (Merino et al. 2016), or bioremediation with the addition of nutrients/inoculants (Kuppusamy et al. 2016). With in situ remediation systems, similar engineered chambers can be found in reactive cells of various types of permeable reactive barriers (Gibert et al. 2007; Davis et al. 2010). The remediation efficiency of ex situ and in situ systems could be improved if they could be coupled with a bio-electrochemical system that would help degradation processes, enhancing the degradation rate and providing an electron acceptor. Here, we propose that the engineered chamber that houses the remediation technology also acts as the bio-electrochemical system (BES) electrode providing an additional mechanism of anaerobic hydrocarbon degradation. This work presents the modification and application of a novel technology called a BES that enhances and monitors biodegradation processes providing an innovative and technically and economically viable sustainable risk management solution (Wang and Ren 2013; Kelly and He 2014).

Bacterial extracellular electron transfer (EET) is a process in which electrons produced through microbial metabolic processes are transferred out of the cell to reduce external solid-state electron acceptors, such as iron (III) oxide, or transferred to conductive minerals such as graphite, and generate energy for growth and/or metabolism. This process has now been identified in a phylogenetically diverse range of environmental bacteria (Lovley 2008) and more species capable of EET are expected to be identified in the future as more research is carried out in this field (Shi et al. 2016; Bjerg et al. 2018; Reguera 2018). The EET process plays an essential role in many biogeochemical cycles and in degradative and natural attenuation processes (Scherr 2013). BESs are engineered environments that manipulate this ability of microbes to oxidize and reduce organic and inorganic matter at an anode and cathode linked via an electronic conductor (Bajracharya et al. 2016). The concept behind a BES is well founded (Logan et al. 2006) with naturally occurring BESs or “biogeobatteries” reported at sites with complex contaminant plumes (Revil et al. 2010; Doherty et al. 2010, 2015). By designing BESs in contaminated groundwater plumes, oxidation and reduction of contaminants or waste substrates are enhanced and limited by the design and emplacement of the electrodes rather than availability of natural electron acceptors in the subsurface. There have been also recent developments of BES for the oxidation of petroleum hydrocarbons (Lu et al. 2014; Daghio et al. 2017; Palma et al. 2018). These approaches use the BES to trigger sulfate-reducing bacteria present in the hydrocarbon-contaminated sediments in an extremely metabolic area, such as changes in the redox environment between contaminated sediments and overlaying water. Many studies have shown also the efficiency of BES in organic-rich sediments with identification of long-distance electron transfer to bridge redox reactions (Daghio et al. 2016; Müller et al. 2016) and effective remediation of complex wastewaters at lab scale (Sevda et al. 2018). Nutrients and heavy metals have also been successfully treated in BES with removal rates up to 70% providing an attractive remediation technology combined with conventional treatment technologies (Zhang et al. 2009, 2014, 2015). Measuring the electrical properties as the output of the BES is a method to monitor the biodegradation activity. The rate of the current production can be used as a proxy for monitoring rates of microbial activity at the field scale (Williams et al. 2010). Williams et al. (2010) measured current densities ranging from 0.2 to ≤ 50 mA/m^2^. The growth of microbes onto the electrodes along with the transfer of electrons can be easily measured using a data-logging voltmeter which allows the system to function as a real-time “biosensor” (ElMekawy et al. 2018) that aids monitoring of microbial activity during remediation. The concept of using BES as biosensors is well understood (Kim et al. 2007; Curtis et al. 2009; Di Lorenzo et al. 2014; Abrevaya et al. 2015) and has mainly been employed to monitor water quality and toxicity (Zhang et al. 2010; Su et al. 2011; Xu and Ying 2011). However, little research has examined the efficiency of such systems using groundwater from complex contaminated plumes such as those associated with gasworks sites. Studies have demonstrated that the exponential phase of biofilm growth matches the exponentially increasing rates of current production measured in the BES (Stein et al. 2012; Bajracharya et al. 2016). Here, degradation and sorption processes are enhanced by the use of a novel engineered graphite BES chamber that contains granular activated carbon (GAC) as high surface area electrodes to trap and degrade organic contaminants. Many studies have shown the application of GAC to sequester organic and inorganic contaminants (Barrow 2012; Mohan et al. 2014). Here, we investigate the use of GAC to enhance the anodic surface of a graphite chamber BES. The effectiveness of GAC as an electrode has already been established (Huggins et al. 2014, 2016). We show that the design of a graphite electrode chamber, coupled with conductive GAC as part of the BES, improves the effectiveness of the conventional non-conductive chambers. Microbial biofilms that anaerobically degrade organic contaminants can pass the resulting electrons directly onto GAC which is a conductive material. It can sorb contaminants rich in light aromatic compounds and, once sorbed, the contaminants can be biodegraded and the resulting electrical output acts as a “remediation sensor.”

## Materials and methods

### Contaminated groundwater sampling

In this study, we used contaminated groundwater dominated by light polycyclic aromatic hydrocarbons (PAHS), such as naphthalene and methyl naphthalenes and BTEX compounds from a former manufactured gas plant at Northern Ireland that was operational for more than 150 years. The operational processes led to contamination of soil and groundwater by coal gasification by-products, which can pose a serious risk to human health and cause significant environmental damage. In the late 1980s, after the production stopped and the plant was closed, the site was remediated and redeveloped. However, the remediation strategy was only applied to shallow subsoil, with contaminants still present in groundwater (at depth > 12 m below surface). Groundwater was sampled from boreholes after purging of three well volumes using a submersible whale pump and stored at 4 °C until use in the experiment.

### BES design and construction

**Fig. 1:**
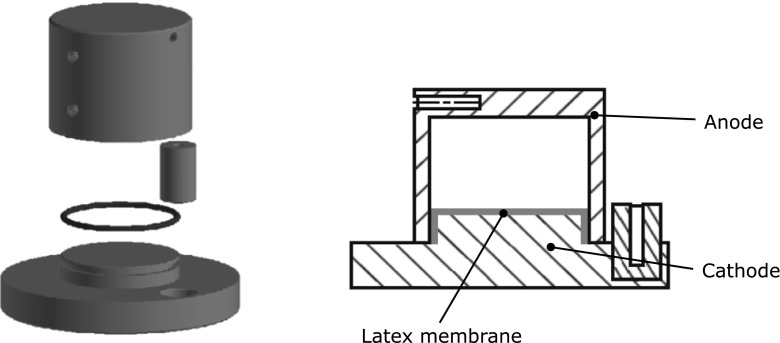
Schematic of the BES and control chambers (all chambers were identical in shape)

One polyethylene terephalate (PET) and four identical graphite chambers were constructed. Each chamber had an external diameter of 5 cm and an internal diameter of 4.1 cm with a volume of 80.46 mL. The chambers of the active BESs also had identical porous graphite base plates; the non-active control chambers had PET base plates separated by a latex membrane. Two graphite chambers were designed to be active BESs; one with GAC designed for water treatment (Jacobi Carbons) and one filled with 3-mm glass beads (SiLibeads, Type M). The glass beads represent non-reactive and non-conductive porous media that could be used in a remediation process as a comparison. Each active BES consisted of one graphite chamber acting as the anode, separated from a porous graphite base plate (cathode) by a latex ion exchange membrane (Winfield et al. 2014a, b). The anode and cathode of the active BES systems were connected by an external wire; the inactive systems were not connected. The three non-active chambers acted as a series of controls to monitor the effectiveness of degradation. The design and configurations can be seen in Fig. 1 and Table 1.

**Table 1:** Experimental setup of chambers used. Non-active BES applies to cells with no cathode connection and no external electron transfer mechanism

Cell	Chamber	Cover	Chamber filling	BES active
1	Graphite	Porous graphite	GAC	Yes
2	Graphite	PET	GAC	No
3	Graphite	Porous graphite	Glass beads	Yes
4	Graphite	PET	Glass beads	No
5	PET	PET	Empty	No

Cell 1 (GAC BES) and cell 3 (glass beads BES) are intended to compare power outputs when the anodic surface is enhanced by a conductive GAC. Comparison of cell 1 (GAC BES) and cell 2 (GAC control) identifies the effect of contaminant decay due to GAC sorption. Comparison of cell 2 (GAC control) and cell 4 (glass control) identifies the sorption capacity of the graphite chambers when glass beads are used as substrate in cell 4. Cell 5 was a PET blank chamber as physiologically inactive material with no graphite present to compare the physical decay of the contaminant. Each cell was connected with a high-precision data-logging Madgetech-Volt101A voltmeter and a variable resistor box. All cells were fed continuously from the same source of contaminated groundwater with the inlet and outlet held in non-reactive Tedlar bags connected with a specialist tubing that helped control the flow rates from peristaltic pumps (Watson Marlow). The flow of the groundwater in the system was kept stable for 3 weeks, that required the flow of 20 L of contaminated groundwater sample from a single container to all the chambers simultaneously in order to maintain the homogeneity of the input. The experiments were carried out under the same conditions (duration, resistor configuration) at room temperature.

### Electrical monitoring

During the experiment, the change of the electrical responses over time was recorded in the BES cells (1 and 3). The cell voltage was measured across an external resistor (Logan et al. 2006) and data logged with a series of voltmeters. The output current calculated with Ohm’s law as *I* = *V*/*R*, where current (*I*) is in amps, voltage (*V*) is in volts, and resistance (*R*) is in ohms, represented through the polarization curves. The cells operated at a constant external resistor load of 10 kΩ. Many studies use 1 kΩ or even less (Zhang et al. 2006; Jadhav and Ghangrekar 2009) for higher current densities; however, 10 kΩ was selected as an attempt to mimic how a larger, field-scale system might operate. In a large-scale BES, significant large potential losses can occur due to higher ohmic resistance of the increased electrode size and surface area, combined with the resistance of wiring and connections (Rozendal et al. 2008). The choice of a very low resistor in field-scale applications can give unrealistic power output evaluations, because of the additional resistance of the wiring and configuration of the system. Low-resistor loads may lead to higher current densities but this can decrease the observed voltage of the system. In this case, the experiment is designed to monitor the output of the system rather than optimize for power. Periodical increase and decrease of the external load occurred at selected days (3, 6, 10, 15, and 21) in order to calculate the polarization curves. The external load was decreased exponentially from 10 ΜΩ until 10 Ω and then back to the higher load every 2 min. The potential of the cell was determined by measuring the voltage against an Ag/AgCl reference electrode with a known potential.

### Bacterial community analysis

To study the bacterial communities, 4 cm^3^ of surface material covered with biofilm was sampled from inside each chamber at the end of the experiment and added to PowerWater® Bead Tubes (MO BIO Lab, Inc.). Microbial DNA was then purified using the MO BIO PowerWater® DNA Isolation Kit and quantified using the QuantiFluor® dsDNA System (Promega). 16S rRNA gene amplicons were generated and sequenced at Molecular Research LP (USA). Briefly, the 16S rRNA gene V4 variable region was amplified using the 515/806 PCR primers (Soergel et al. 2012), with a barcode on the forward primer. A 30-cycle PCR was performed using the HotStarTaq Plus Master Mix Kit (Qiagen, USA) at the following cycling conditions: 95 °C for 5 min and 28 cycles of 94 °C for 30 s, 53 °C for 40 s, 72 °C for 1 min, and a final extension of 72 °C for 5 min. After amplification, PCR products were checked after electrophoresis in a 2% agarose gel and samples were pooled together in equal proportions based on their molecular weight and concentration. Afterwards, PCR products were purified with calibrated AMPure® XP Beads (Beckman Coulter, Inc.) and used to prepare the DNA library following the Illumina® TruSeq DNA library protocol. Samples were sequenced on the Illumina®MiSeq System. Generated read pairs were joined after q25 trimming on both ends and quantitative sequence analysis was carried out using QIIME 1.9.1 (Caporaso et al. 2011). USEARCH v6.1.544 (Edgar 2010) was used to assign Operational Taxonomic Units (OTUs) based on 97% similarity with a de novo method. Singletons were removed during the process. Alignment of sequences was done using PyNAST (Caporaso et al. 2010) and taxonomy was assigned to sequences using the most recent Greengenes reference database (DeSantis et al. 2006) (released on August 2013) with the UCLUST algorithm (Edgar 2010). The produced BIOM table was rarefied using the lowest sample count for normalization of data and uploaded to Calypso (Zakrzewski et al. 2017) for downstream analysis. Top taxa were selected followed by removal of rare results (≤ 0.001 relative abundance across all samples). Evenness and richness indexes were used to estimate alpha diversity of the samples and top taxa were selected for quantitative taxonomic analysis. Multivariate analysis (PCoA) of the samples was performed using the Unweighted UniFrac method before removal of rare counts.

### Chemical analysis

Groundwater from each of the cells at the end of the experiment was analyzed using 2-dimensional gas chromatography with a flame ionization detector (GCxGC FID). 2D gas chromatography (GCxGC) is a powerful tool for environmental analysis of organic compounds which splits the sample across two GC columns allowing information about retention time and polarity of the sample with one injection reducing analysis time (Welke and Zini 2011). GCxGC-FID analysis of the groundwater was performed using an Irish National Accreditation Board (INAB accredited) Total Petroleum Hydrocarbon Criteria Working Group (TPHCWG) method by Complete Laboratory Solutions (CLS) in RosMuc, Galway, Ireland, to group them in terms of risk management.

## Results and discussion

### Electrical monitoring

**Fig. 2:**
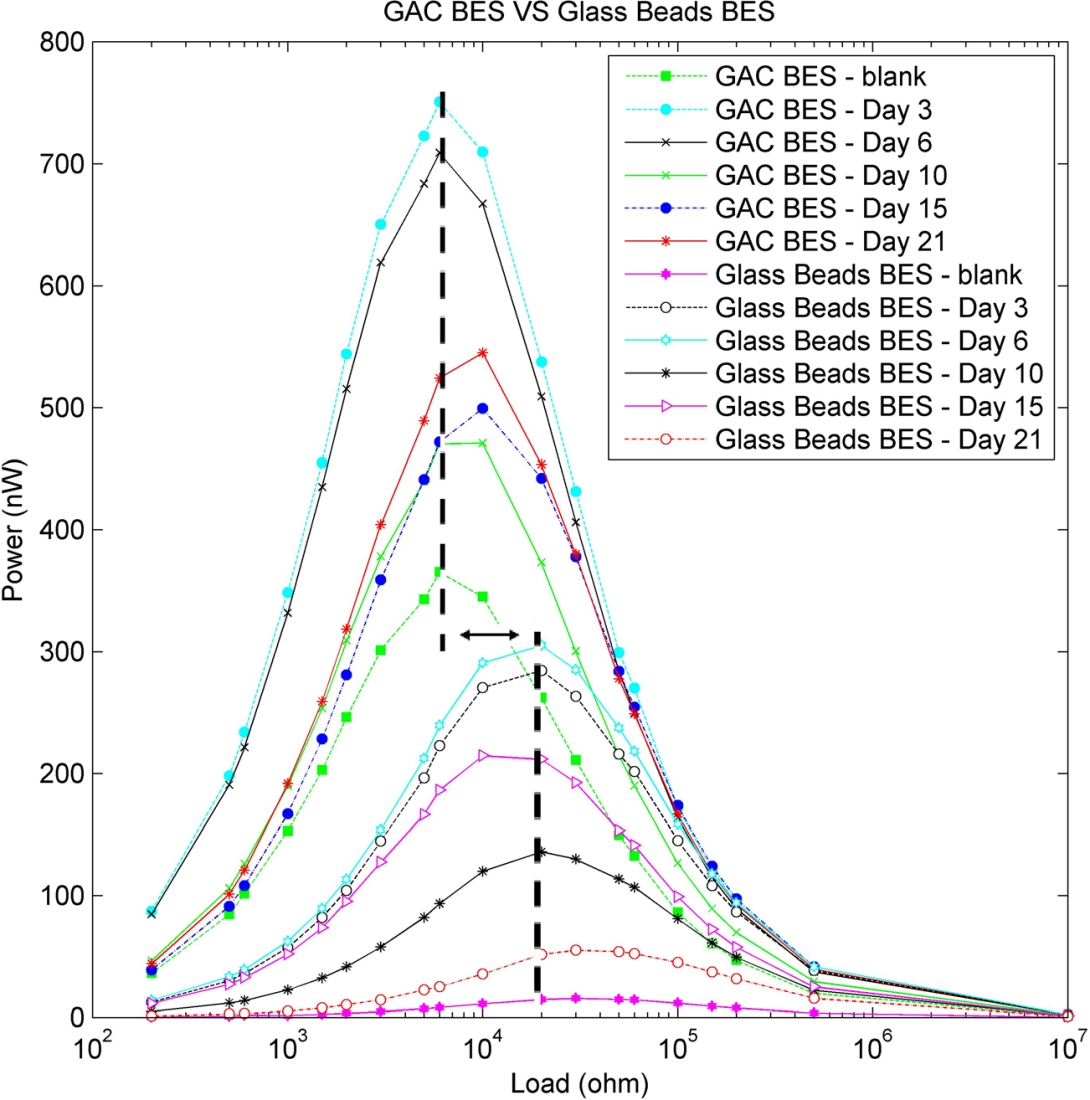
Power curve between GAC BES and glass beads BES during the treatment process. There is notable offset on the internal resistance between GAC BES and glass beads BES and is due to higher surface area of the GAC BES electrode

**Fig. 3:**
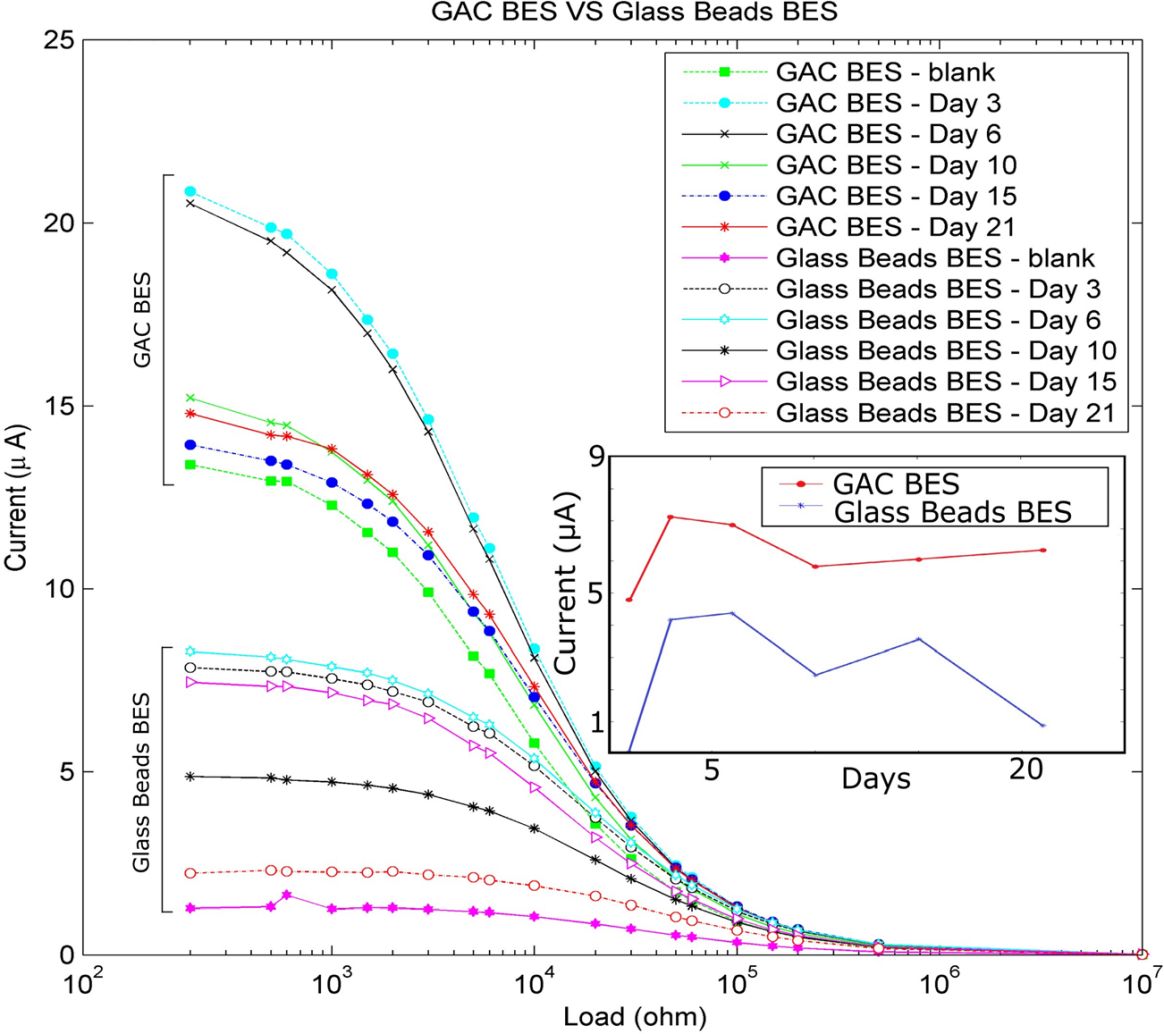
Current curve between GAC BES and glass beads BES during the treatment process. The GAC BES occupies the top half of the image with the output from the glass beads BES in the bottom half of the image. Inset shows current output over time

In Figs. 2 and 3, the calculated power and current outputs respectively during the measurements across a wide range of resistance options are presented. The GAC BES was more effective than the glass beads BES in terms of power and current output that can be related with the higher efficiency of the GAC BES. Since the polarization curve in the preliminary results peaks at about 10–100 kΩ, there was no need to use very low resistor options as we were focused on the degradation capacity of the system rather than the power output. In Fig. 2, there is a clear offset on the ohm axis (X-axis) between GAC BES and glass beads BES that is related to the higher surface area of GAC. The larger surface area may be responsible for the lower resistivity of the load for the GAC BES compared with the glass beads BES. Several studies have demonstrated the increased electrical response (current and power generation) of BES systems when they are enhanced with high conductivity and high surface area electrodes (Liang et al. 2011; Li et al. 2014; Liu et al. 2014; Ge et al. 2015; Tursun et al. 2016). In our example, the GAC BES’s increased response is enhanced by the additional surface area of the GAC acting as the anode within the graphite chamber. The average current output of the GAC BES was 10–20 μV to a chamber of 80 cm^3^. This is equal to an average current output of 0.78 μA/cm^2^ or 7.8 mA/m^2^ which is in agreement with standard BES applications and a current output of 0.2 to 2 μA/cm^2^ (Bretschger et al. 2010; Lu et al. 2017). Studies have demonstrated also average current production 10 mA/m^2^ and 25 mA/m^2^ in freshwater and marine sediment BES, respectively (Tender et al. 2002; Holmes et al. 2004).

It can be seen that the electrical responses, including current generation (Fig. 3), increase from the injection of contaminated groundwater, and reaches a maximum after 3 days. The responses then showed a drop and offset in the measured current production after 6 days of treatment, followed by approximately stable measurements over the rest of the experiment; this indicates that the maximum growth and electroactivity of the microbial community occurred in the first week of the BES operation. This suggests that the BESs are rapidly colonized with both degrading and electroactive microbes. Scaling up of the system can produce a strong redox environment, easily measured by electrical geophysical methods (Doherty et al. 2010).

### Bacterial community analysis

**Fig. 4:**
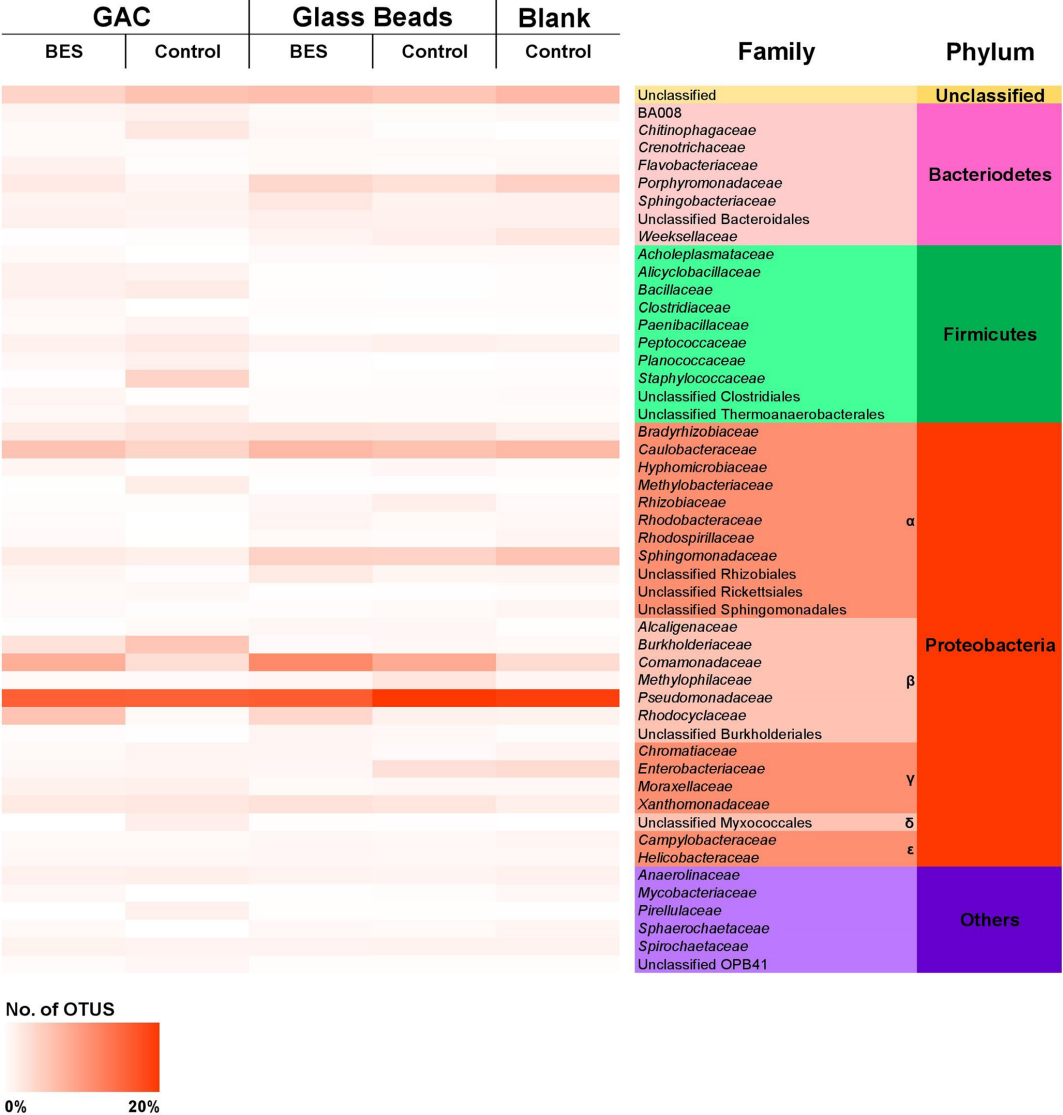
Bacterial taxonomic diversity. Top 50 families represented. Proteobacteria and specifically β-proteobacteria (particularly *Psuedomondaceae*) dominate all the samples. The bacterial families from the blank control are considered representative of the contaminated groundwater and these families are similarly abundant in BESs and control experiments

**Fig. 5:**
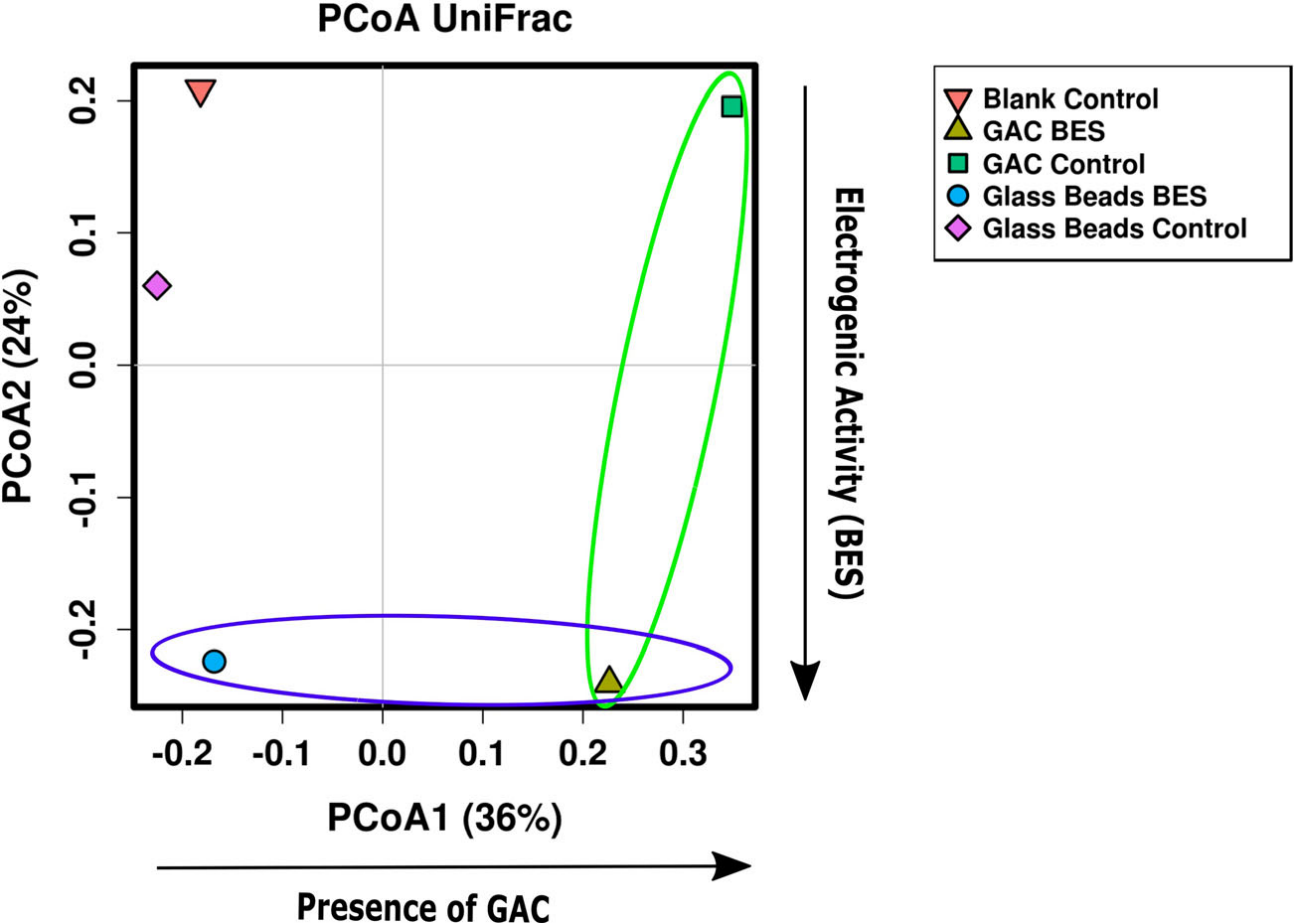
Principal coordinate UniFrac analysis of the cells. Principal coordinate 1 (x-axis) is controlled by the presence or absence of GAC with positive values representing the presence of GAC (GAC present in green ellipse). Principal coordinate 2 (y-axis) is controlled by the presence or absence of electrogenic activity with negative values representing the presence of the bio-electrochemical systems (BES active in blue ellipse)

Twenty-six phyla of bacteria were found. Proteobacteria, Bacteroidetes, and Firmicutes were the most abundant phyla identified in the experiments. Proteobacteria were particularly represented by its gamma, beta, and alpha classes. Overall, 53 classes of bacteria were found. Other classes found in the experiments included Bacilli and Bacteroidia. The analysis of the bacterial taxonomic diversity at phylum and family levels can be observed in the heat map of Fig. 4. The results show that Proteobacteria and specifically β-proteobacteria (particularly *Psuedomondaceae*) dominate all the samples. The bacterial families from the blank control are considered representative of the contaminated groundwater and these families are similarly abundant in BESs and control experiments. This may be due to the relatively short time frame of the experiment (21 days). It is interesting that the BESs were still able to provide an increase in electrical output relatively quickly suggesting that the microbial communities present in the contaminated groundwater are well suited for both degradation and electron transfer. *Pseudomonadaceae* contain aerobic degraders and could be expected not to dominate within an anaerobic groundwater sample or the anaerobic anode of a BES, but here it dominates all samples occurring at 19% in the blank and glass beads controls and ~ 16% in the two active BES and GAC control samples. *Pseudomonas* spp. may have evolved to be active degraders under reducing conditions and some *Pseudomonas* spp. can utilize electron shuttles to transfer electrons to an anode (Daghio et al. 2017). This may be the case here with only a minor reduction in *Psuedomondaceae* (~ 3%) observed in the active BESs compared with the blank control groundwater*. Rhodocyclaceae* showed an increase in the active BES chambers (6% in GAC and 4% in the glass beads) when compared with the blank groundwater control (1%). A similar pattern was observed with *Comamonadaceae*, with blank control having 3% of OTUs and the glass beads BES and GAC BES having 12% and 8% respectively. *Rhodocyclaceae* and *Comamonadaceae* are known as degraders (Singleton et al. 2015) and are commonly found in bio-electrochemical systems (Timmers et al. 2012). *Caulobacteraceae*, which is a family of the α-proteobacteria phyla that contain Fe(III) reducers, occurs from 4 to 7% in all samples (Li et al. 2013). Such Fe(III) reducers including *Burkholderiaceae* and S*phingomonadaceae*, which are also found across all samples, may also be active in the transfer of electrons and enhanced anaerobic oxidation of organic contaminants (Lovley 1997). The amount of unclassified OTUs, between 4 and 7% in all samples, also suggests the possible presence of unrecognized electrogenic and degrading species. Overall, a total of 143 families and 209 genera were identified in BESs and control experiments. At the genus level, only 108 OTUs were classifiable using the most recent Greengenes database (DeSantis et al. 2006); however, abundant genera found included *Pseudomonas*, *Sphingobium*, *Burkholderia*, *Novosphingobium*, and *Comamonas* species. Principle coordinate analysis of UniFrac OTUs, which considers all OTUs not just those shown in Fig. 4, shows that the blank control and glass beads control are distinct from the other BES and GAC formations (Fig. 5). The variance on the X-axis (36%) is related to the presence of GAC, with the non-GAC controls and non-GAC BES plotting with negative values and the GAC control and GAC BES plotting with positive values. The Y-axis (24% of variance) is attributed to electrogenic activity with the two active BES plotting with negative values and all three non-active controls plotting with positive values. This suggests that, despite the top families being found at similar ratios across all samples, even after a relatively short experimental time (21 days) the microbial communities are successfully adapting to electroactive and high-sorbing environments (OTU level). This may be due to the fact that the gasworks-contaminated groundwater comes from a site that had operated for over 100 years and the microbial communities present are well adapted to the high levels of contamination and changing redox environments that could be expected across older groundwater plumes (Meckenstock et al. 2015). Such older microbial communities that exist around contaminated environments for example those around steady state or reducing plumes may be more readily adapted to the BES technologies.

### Chemical analysis

Table 2 shows the results of the GCxGC-FID analysis on the contaminated groundwater post chamber. The blank control contained high levels of aliphatic and aromatic hydrocarbons (total TPH 1546 mg/L). The majority of the contamination was in the lighter aromatic fractions (C10–C14), which is characteristic of a carbureted water gas (CWG) process (Gallacher et al. 2017). The GAC BES gave the best removal performance (99% removal) overall followed by the GAC control (97% removal). The glass beads control and the glass beads BES removed 40 and 32% of the TPH, respectively; this removal may be due to the graphite of the chamber also sorbing the organic contaminants. The GAC BES and GAC control both show high hydrocarbon removal over a short experimental time (21 days) with the GAC BES improving overall removal performance of the control GAC by 2% with a 99.04% reduction compared with 97.83%. Critically, the GAC BES outperforms the GAC control and the others in the aromatic C10–C12 fraction which accounts for the majority of the contaminant load (81.1%). Here, we see increased aromatic degradation that is probably related to anaerobic biodegradation processes associated with naphthalene and methylnaphthalene removal. The pathways for anaerobic degradation of naphthalene compounds as found on gasworks sites have been described in detail (Griebler et al. 2004). Often, the aromatic compounds are oxidized to organic acids which are in turn degraded to long-chain fatty acids and these are finally metabolized into methane and carbon dioxide (Abbasian et al. 2015; Varjani 2017). It has recently been suggested that *Pseudomonas* spp. have evolved to be active degraders under reducing conditions and some *Pseudomonas* spp. can utilize electron shuttles to transfer electrons to an anode (Daghio et al. 2017) as may be the case here. This suggests that, as expected, the best removal performance is dominated by a quick sorption mechanism followed by degradation within the GAC BES. However, the outperformance in reduction of contaminant load of the GAC BES compared with the glass beads BES suggests that an inert non-conductive matrix within the BES chamber does not provide an efficient substrate for the BES to be used as the sole method of contaminant reduction of PAHs from a gasworks source. The negative percent reduction of the glass beads cells in the aliphatic fraction C10–C12 indicates the breakdown and cleavage of the aromatic fraction into the aliphatic fraction which has yet to degrade. The heavy aromatic fraction (C35–C44) was not removed as well as the other hydrocarbon fractions (48–56%) but this fraction accounted for only 0.56% of the total contaminant load.

**Table 2:** Aliphatic and aromatic hydrocarbon concentrations in mg/L. Each chamber is compared with the blank control to determine the additional percent reduction of contaminants compared to the natural reduction

TPH CWG	Blank control	GAC BES	Reduction (%)	GAC control	Reduction (%)	Glass beads BES	Reduction (%)	Glass beads control	Reduction (%)
Aliphatics nC8-nC10	18.33	0.21	98.84	0.35	98.09	4.8	73.81	13.65	25.53
Aliphatics > nC10–nC12	21.17	0.68	96.8	1.82	91.40	65.94	− 211.48	33.9	− 60.13
Aliphatics > nC12–nC16	40.14	0.24	99.4	0.44	98.90	15.05	62.51	8.5	78.82
Aliphatics > nC16–nC21	4.08	0.03	99.24	0.17	95.83	0.4	90.20	0.32	92.16
Aliphatics > nC21–nC35	9.65	1.27	86.84	1.88	80.52	2.03	78.96	1.77	81.66
Aliphatics > nC35–nC44	2.61	0.17	93.34	0.43	83.52	0.42	83.91	0.3	88.51
Aromatics eC8–eC10	22.5	0.66	97.05	1.68	92.53	12.49	44.49	11.68	48.09
Aromatics > eC10–eC12	1255.1	5.74	99.54	19.2	98.47	860.69	31.42	779.69	37.88
Aromatics > eC12–eC16	131.55	0.67	99.49	1.52	98.84	70.88	46.12	54.01	58.94
Aromatics > eC16–eC21	22.21	0.03	99.86	0.36	98.38	8.3	62.63	5.61	74.74
Aromatics > eC21–eC35	8.6	0.25	97.08	0.34	96.05	0.46	94.65	0.38	95.58
Aromatics > eC35–eC44	10.52	4.82	54.17	5.4	48.67	4.8	54.37	4.63	55.99
Total aliphatics	95.98	2.6	97.29	5.1	94.69	88.63	7.66	58.43	39.12
Total aromatics	1450.48	12.18	99.16	28.51	98.03	957.62	33.98	856	40.99
Total Aliph.–aromatics	1546.45	14.78	99.04	33.6	97.83	1046.25	32.35	914.42	40.87

## Conclusions

The electrical output from the GAC BES was greater than the glass beads BES due to the greater available surface area of the GAC that also acted as the anode electrode. The electrical response of the GAC BES, by power or current output, can be used as management tool or proxy to monitor microbial activity. A higher flow of electrons reflects indirectly a more metabolically active community that is a well-established phenomenon, and it was shown here. However, this is an indirect measure of microbial activity. The electrical response, hand in hand with the removal of contaminants, poses a strong case in the clarification of the microbial activity in the systems studied. Bacterial community analysis shows that β-proteobacteria dominate all the samples particularly the PAH-degrading *Psuedomondaceae* family, and *Rhodocyclaceae* and *Comamonadaceae* OTU families are observed to increase in BES cells. PCoA of UniFrac Observed Taxonomic Units shows distinct grouping of microbial types that are associated with the presence of GAC, and grouping of microbial types associated with electroactivity. This combined with the electrical output of the BES suggests that BES chambers are quickly colonized and optimized by the indigenous microbial communities found in gasworks-contaminated groundwater. The GAC BES was the most effective in removing total petroleum hydrocarbon contamination from the gasworks-contaminated groundwater (99% removal). The GAC BES performs better than the GAC control, with the GAC BES removing more light aromatic compounds that dominate the contaminant load. This may prove to be a valuable practical application where additional biological removal of contaminants of concern is required. The glass beads control has an intriguing higher decay rate compared with the glass beads BES. In such a low conductivity medium, like glass beads, the BES appears to not work properly and its results are equivalent to the glass beads control cell. The GAC BES is a good candidate as a sustainable remediation technology that provides improved degradation over GAC and near real-time observation of associated electrical output. Future work around the analysis of the different types of GAC, i.e., surface area as a controlling factor in the power/current generated vs sorption capacity in the BES units, should also be considered. Further work should consider the longer term performance of the system focusing on the degradation of hydrocarbons that have sorbed to the GAC in the BES chamber.

## Electronic supplementary material

ESM 1 (DOCX 195 kb)
