# Supplementary material for: Enhancement of gasworks groundwater remediation by coupling a bio-electrochemical and activated carbon system
Source: Environ Sci Pollut Res Int. 2019 Feb 9;26(10):9981–91. doi: 10.1007/s11356-019-04297-w (PMC6469603; doi:10.1007/s11356-019-04297-w)
Supplement: Supplementary file 1 — (DOCX 195 kb) [file 11356_2019_4297_MOESM1_ESM.docx]

**Supplementary Material**

Enhancement of Gasworks Groundwater Remediation by coupling a Bio-electrochemical and Activated Carbon System

*Panagiotis Kirmizakis^a^, Rory Doherty^a*^, Carlos A. Mendonça^b^, Ricardo Costeira^c^, C.C.R. Allen^c,d^, Ulrich S. Ofterdinger^a^, Leonid Kulakov^c^*

^a^ School of the Natural and Built Environment, Queen’s University Belfast, Stranmillis Road, BT9 5AG, Belfast, United Kingdom

^b^ University of São Paulo, Department of Geophysics, University of São Paulo, Rua do Matão, 1226, São Paulo, Brazil

^c^ School of Biological Sciences, Queen’s University Belfast, Lisburn Road, BT9 7BL, Belfast, United Kingdom

^d^ Institute for Global Food Security, Queen’s University Belfast, Lisburn Road, BT9 7BL, Belfast, United Kingdom

*Corresponding author

**
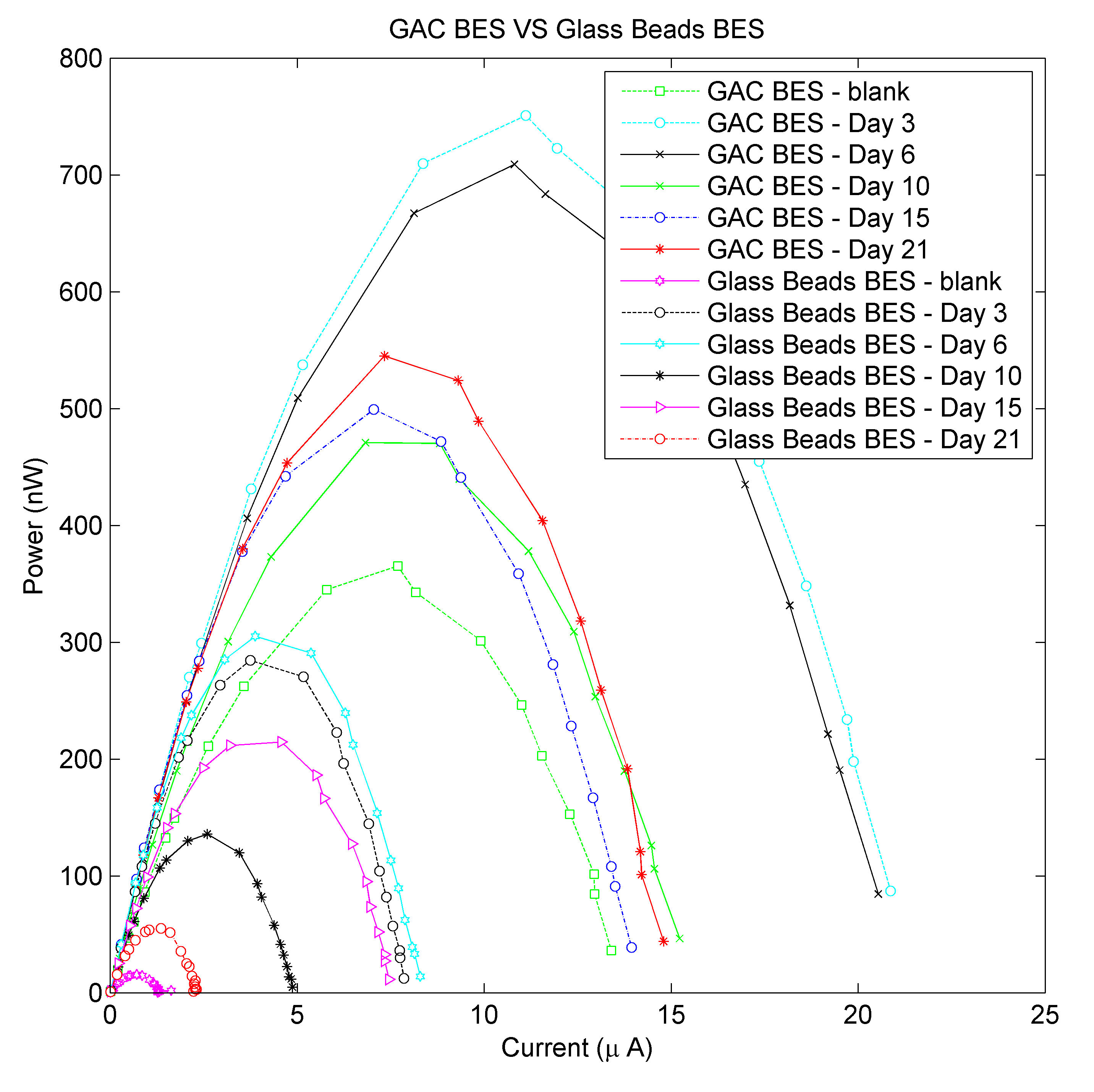
**

**Supplementary Figure 1** Power density Vs Current density relationship for Granular Activated Carbon (GAC) Bio-Electrochemical System (BES) and Glass Beads BES during the treatment process. The GAC BES was more effective than the Glass Beads BES in terms of power and current output. There is notable offset between GAC BES and Glass Beads BES with stabilization of the GAC BES output between 10 and 21 days.

**Supplementary Table 1** Raw data of bacterial taxonomic diversity (143 families represented).

| **GAC BES** | **GAC Control** | **Glass Beads BES** | **Glass Beads Control** | **Teflon Control** |  |
| --- | --- | --- | --- | --- | --- |
| 0.16000164 | 0.158295493 | 0.163503114 | 0.199281592 | 0.19274912 | Pseudomonadaceae |
| 0.0818768 | 0.033739168 | 0.117913546 | 0.085078318 | 0.036185606 | Comamonadaceae |
| 0.060164635 | 0.042612679 | 0.070500396 | 0.061912613 | 0.070777486 | Caulobacteraceae |
| 0.044664733 | 0.061747735 | 0.065167276 | 0.058732776 | 0.071006799 | Unclassified |
| 0.019979703 | 0.01529813 | 0.045337248 | 0.0447886 | 0.060538656 | Sphingomonadaceae |
| 0.021158597 | 0.009349408 | 0.039430675 | 0.030396891 | 0.047720055 | Porphyromonadaceae |
| 0.059426545 | 0.006940176 | 0.040336732 | 0.014085502 | 0.012405838 | Rhodocyclaceae |
| 0.019979703 | 0.027542583 | 0.02860387 | 0.027193499 | 0.015341046 | Bradyrhizobiaceae |
| 0.021384125 | 0.023378478 | 0.028993818 | 0.025532917 | 0.015765275 | Xanthomonadaceae |
| 0.028724026 | 0.058128929 | 0.005505155 | 0.007537393 | 0.006489561 | Burkholderiaceae |
| 0.008457288 | 0.010588725 | 0.008578867 | 0.03106819 | 0.035635255 | Enterobacteriaceae |
| 0.011778696 | 0.012343598 | 0.023224874 | 0.012896008 | 0.014504053 | Sphingobacteriaceae |
| 0.013634174 | 0.021217109 | 0.011354383 | 0.01596985 | 0.012509029 | Peptococcaceae |
| 0.014054475 | 0.009864964 | 0.015552064 | 0.013732187 | 0.014951214 | Unclassified Bacteroidales |
| 0.014167239 | 0.015456763 | 0.011297038 | 0.009351078 | 0.014641641 | Anaerolinaceae |
| 0.012260505 | 0.011520692 | 0.011182347 | 0.013319986 | 0.013277228 | Spirochaetaceae |
| 0.002296282 | 0.002429061 | 0.011411728 | 0.017630432 | 0.0253047 | Weeksellaceae |
| 0.006407036 | 0.00524479 | 0.009737244 | 0.025662466 | 0.008736829 | Methylophilaceae |
| 0.002296282 | 0.043941227 | 0.00198415 | 0.003120952 | 0.00324478 | Staphylococcaceae |
| 0.008939097 | 0.00343043 | 0.02166508 | 0.009645507 | 0.009596753 | Unclassified.Rhizobiales |
| 0.01409548 | 0.015070096 | 0.005963918 | 0.00725474 | 0.007166034 | Moraxellaceae |
| 0.010845831 | 0.014871805 | 0.006583248 | 0.004993523 | 0.007601729 | BA008 |
| 0.006232765 | 0.009349408 | 0.010058378 | 0.005393947 | 0.011752296 | Chromatiaceae |
| 0.006478795 | 0.02310087 | 0.008269202 | 0.003120952 | 0 | Chitinophagaceae |
| 0.01360342 | 0.01958121 | 0.001617139 | 0.001177718 | 0.002809085 | Bacillaceae |
| 0.015407641 | 0.017053003 | 0.00198415 | 0.002638087 | 0.001146565 | Unclassified Streptophyta |
| 0.006642815 | 0.00642462 | 0.008028352 | 0.007902485 | 0.00902347 | Campylobacteraceae |
| 0.007462916 | 0.007009577 | 0.008028352 | 0.007725827 | 0.007521469 | Helicobacteraceae |
| 0.007606434 | 0.015427019 | 0.004140336 | 0.004852196 | 0.004999025 | Unclassified Thermoanaerobacterales |
| 0.002716583 | 0.002805814 | 0.008028352 | 0.017865976 | 0.005503514 | Rhizobiaceae |
| 0.014679802 | 0.002429061 | 0.00668647 | 0.004557767 | 0.007945699 | Flavobacteriaceae |
| 0.013408646 | 0.011044794 | 0.001617139 | 0.002355435 | 0.00324478 | Alicyclobacillaceae |
| 0.006150755 | 0.004649918 | 0.00606714 | 0.006972088 | 0.006581286 | Crenotrichaceae |
| 0.005709951 | 0 | 0.007168171 | 0.006866094 | 0.010192967 | Sphaerochaetaceae |
| 0.004582312 | 0 | 0.009737244 | 0.006760099 | 0.007773714 | Rhodobacteraceae |
| 0.005422916 | 0.000991454 | 0.006789692 | 0.004074903 | 0.010250295 | Rhodospirillaceae |
| 0.005330654 | 0.003132994 | 0.003245748 | 0.006665882 | 0.00902347 | Unclassified Sphingomonadales |
| 0.008754575 | 0.00139795 | 0.003624227 | 0.008326463 | 0.004861438 | Hyphomicrobiaceae |
| 0.007534675 | 0.013523428 | 0.002293815 | 0 | 0.002293131 | Planococcaceae |
| 0.003833971 | 0.00139795 | 0.0095308 | 0.007808268 | 0.002568307 | Unclassified Burkholderiales |
| 0.001445427 | 0.005522397 | 0.008188919 | 0.008326463 | 0.001616657 | Alcaligenaceae |
| 0.00945166 | 0.001715215 | 0.004140336 | 0.004404664 | 0.005377392 | Unclassified Clostridiales |
| 0.006642815 | 0 | 0.005505155 | 0.006760099 | 0.005847484 | Acholeplasmataceae |
| 0.001025126 | 0.018331978 | 0.001146907 | 0.002037451 | 0.001983558 | Methylobacteriaceae |
| 0 | 0.017340525 | 0.00198415 | 0.002355435 | 0 | Unclassified Myxococcales |
| 0.005884222 | 0.007872142 | 0.002293815 | 0.002355435 | 0.00324478 | Unclassified Rickettsiales |
| 0.004695076 | 0.008120006 | 0.002809923 | 0.003120952 | 0.002809085 | Unclassified OPB41 |
| 0.007944725 | 0.000991454 | 0.003245748 | 0.003721588 | 0.003806597 | Clostridiaceae |
| 0.007667941 | 0 | 0.001146907 | 0.003120952 | 0.007166034 | Mycobacteriaceae |
| 0.005525428 | 0.011213341 | 0.001146907 | 0 | 0.001146565 | Paenibacillaceae |
| 0 | 0.01422736 | 0.001617139 | 0.001660582 | 0.001146565 | Pirellulaceae |
| 0.001025126 | 0.00139795 | 0.004862887 | 0.004852196 | 0.006283179 | Unclassified Mollicutes |
| 0.001025126 | 0.000991454 | 0.001146907 | 0.004852196 | 0.010387883 | Isosphaeraceae |
| 0 | 0 | 0.00606714 | 0.005393947 | 0.006879393 | ML635J40 |
| 0.001773468 | 0 | 0.003440722 | 0.004074903 | 0.00857631 | Dehalococcoidaceae |
| 0.004695076 | 0.009666673 | 0.002293815 | 0.001177718 | 0 | Lachnospiraceae |
| 0.008139499 | 0.007931629 | 0.001146907 | 0 | 0 | Propionibacteriaceae |
| 0.002296282 | 0.000991454 | 0.003624227 | 0.004074903 | 0.00596214 | Desulfobulbaceae |
| 0.003403418 | 0.001715215 | 0.003968299 | 0.004557767 | 0.00324478 | Unclassified Betaproteobacteria |
| 0 | 0.014405822 | 0.001146907 | 0 | 0.001146565 | Unclassified WCHB107 |
| 0.007391157 | 0.000991454 | 0.004140336 | 0.002355435 | 0.001616657 | Oxalobacteraceae |
| 0.001773468 | 0.010449922 | 0.001146907 | 0.001660582 | 0.001146565 | Coxiellaceae |
| 0.004100503 | 0.009141203 | 0 | 0.001660582 | 0.001146565 | Micrococcaceae |
| 0.012045229 | 0.000991454 | 0.001146907 | 0 | 0.001616657 | Unclassified OP112 |
| 0 | 0.000991454 | 0.001617139 | 0.005393947 | 0.007773714 | Microbacteriaceae |
| 0.002901106 | 0 | 0.003968299 | 0.004993523 | 0.003623147 | Tissierellaceae |
| 0.001445427 | 0.000991454 | 0.003968299 | 0.004074903 | 0.003806597 | Carnobacteriaceae |
| 0 | 0.011520692 | 0.001146907 | 0 | 0.001616657 | At425_EubF1 |
| 0.005709951 | 0.003965815 | 0 | 0.001177718 | 0.001616657 | Pelobacteraceae |
| 0.002511558 | 0.001715215 | 0.002569072 | 0.002638087 | 0.002809085 | Unclassified OP3 |
| 0.001445427 | 0.006345303 | 0.00198415 | 0.001177718 | 0.001146565 | Hydrogenophilaceae |
| 0 | 0.009666673 | 0 | 0.001177718 | 0.001146565 | Syntrophaceae |
| 0.002716583 | 0.009250263 | 0 | 0 | 0 | Brucellaceae |
| 0.009226133 | 0.000991454 | 0 | 0 | 0.001616657 | Xanthobacteraceae |
| 0.001025126 | 0.000991454 | 0.004289434 | 0.002037451 | 0.003439696 | Bacteroidaceae |
| 0 | 0.007148381 | 0.002293815 | 0.001177718 | 0.001146565 | Erysipelotrichaceae |
| 0.002050252 | 0.000991454 | 0.003039305 | 0.002638087 | 0.003038398 | Desulfobacteraceae |
| 0 | 0.008298467 | 0 | 0.001660582 | 0.001146565 | Sinobacteraceae |
| 0.003833971 | 0.000991454 | 0.002293815 | 0.002355435 | 0.001616657 | Acetobacteraceae |
| 0 | 0.009666673 | 0.001146907 | 0 | 0 | Unclassified TM71 |
| 0.001445427 | 0 | 0.003039305 | 0.003120952 | 0.002809085 | Synergistaceae |
| 0.004920604 | 0.003836926 | 0 | 0.001177718 | 0 | Corynebacteriaceae |
| 0.001025126 | 0.003132994 | 0.001146907 | 0.002037451 | 0.002568307 | Unclassified KD3145 |
| 0 | 0 | 0.00198415 | 0.001660582 | 0.006179988 | Procabacteriaceae |
| 0.009564424 | 0 | 0 | 0 | 0 | Trueperaceae |
| 0.002050252 | 0.001715215 | 0.001617139 | 0.002037451 | 0.001983558 | Unclassified ZB2 |
| 0.001445427 | 0 | 0.00198415 | 0.002037451 | 0.003806597 | R445B |
| 0 | 0 | 0.001146907 | 0.007984925 | 0 | Planctomycetaceae |
| 0.001025126 | 0 | 0.002809923 | 0.002638087 | 0.002568307 | Ruminococcaceae |
| 0.003700704 | 0 | 0.001146907 | 0.001177718 | 0.002568307 | Veillonellaceae |
| 0.001445427 | 0.001715215 | 0.001617139 | 0.002638087 | 0.001146565 | Unclassified.BD49 |
| 0.002716583 | 0.00139795 | 0.00198415 | 0.002355435 | 0 | Eubacteriaceae |
| 0.004223518 | 0.000991454 | 0.001146907 | 0 | 0.001983558 | RFP12 |
| 0.001025126 | 0.000991454 | 0.001146907 | 0.002885408 | 0.001983558 | Cloacamonaceae |
| 0.003967237 | 0 | 0.001617139 | 0.001177718 | 0.001146565 | Erythrobacteraceae |
| 0.005330654 | 0.000991454 | 0 | 0.001177718 | 0 | Unclassified Thermoleophilia |
| 0.001025126 | 0.000991454 | 0 | 0.002355435 | 0.002809085 | Bacteriovoracaceae |
| 0.005884222 | 0 | 0.001146907 | 0 | 0 | Unclassified.BD73 |
| 0.006724825 | 0 | 0 | 0 | 0 | Unclassified RB41 |
| 0.001025126 | 0 | 0.00198415 | 0.001177718 | 0.001983558 | Nocardiaceae |
| 0 | 0 | 0.001617139 | 0.001660582 | 0.002809085 | Shewanellaceae |
| 0.001025126 | 0.004957268 | 0 | 0 | 0 | Syntrophorhabdaceae |
| 0.005802212 | 0 | 0 | 0 | 0 | Kineosporiaceae |
| 0 | 0.000991454 | 0.001617139 | 0.002037451 | 0.001146565 | Syntrophomonadaceae |
| 0 | 0.005690944 | 0 | 0 | 0 | Aeromonadaceae |
| 0.001025126 | 0 | 0.001146907 | 0.001177718 | 0.002293131 | Unclassified PBS25 |
| 0 | 0 | 0.001617139 | 0.002037451 | 0.001983558 | Dehalobacteriaceae |
| 0.001025126 | 0.00139795 | 0 | 0.002037451 | 0.001146565 | Unclassified OD1 |
| 0 | 0 | 0 | 0 | 0.005503514 | Verrucomicrobiaceae |
| 0.004469549 | 0.000991454 | 0 | 0 | 0 | Gaiellaceae |
| 0.001773468 | 0.00343043 | 0 | 0 | 0 | Micromonosporaceae |
| 0 | 0 | 0.001617139 | 0.001177718 | 0.002293131 | ML1228J1 |
| 0 | 0 | 0.001146907 | 0.001177718 | 0.002293131 | Phyllobacteriaceae |
| 0 | 0 | 0.001617139 | 0 | 0.002809085 | Puniceicoccaceae |
| 0.001025126 | 0.000991454 | 0.001146907 | 0.001177718 | 0 | kpj58rc |
| 0 | 0 | 0.001617139 | 0 | 0.002293131 | Unclassified.GIF9 |
| 0.001025126 | 0 | 0.001617139 | 0.001177718 | 0 | Unclassified.SJA4 |
| 0 | 0 | 0.003807732 | 0 | 0 | Unclassified Stramenopiles |
| 0 | 0.000991454 | 0 | 0.001177718 | 0.001616657 | Unclassified ML615J28 |
| 0 | 0 | 0 | 0.002037451 | 0.001616657 | Ellin6075 |
| 0 | 0 | 0 | 0.001660582 | 0.001983558 | Microthrixaceae |
| 0 | 0 | 0.001146907 | 0.002355435 | 0 | Hyphomonadaceae |
| 0.001773468 | 0.001715215 | 0 | 0 | 0 | Methylococcaceae |
| 0 | 0 | 0 | 0.001177718 | 0.002293131 | Unclassified ML635J21 |
| 0.001025126 | 0 | 0.001146907 | 0.001177718 | 0 | Unclassified PL11B10 |
| 0 | 0 | 0.001617139 | 0.001660582 | 0 | Unclassified WS1 |
| 0.002050252 | 0 | 0 | 0 | 0.001146565 | X429 |
| 0 | 0 | 0.00198415 | 0 | 0.001146565 | Thermodesulfovibrionaceae |
| 0.003075378 | 0 | 0 | 0 | 0 | Unclassified MIZ46 |
| 0.002901106 | 0 | 0 | 0 | 0 | Koribacteraceae |
| 0 | 0 | 0 | 0.001660582 | 0.001146565 | Unclassified Actinomycetales |
| 0 | 0 | 0 | 0.001660582 | 0.001146565 | Methylocystaceae |
| 0 | 0 | 0 | 0.001660582 | 0.001146565 | Alcanivoracaceae |
| 0 | 0 | 0 | 0.001177718 | 0.001616657 | Desulfovibrionaceae |
| 0 | 0 | 0.001617139 | 0 | 0.001146565 | Unclassified mle18 |
| 0.002716583 | 0 | 0 | 0 | 0 | Unclassified.Gemm1 |
| 0 | 0.000991454 | 0 | 0.001660582 | 0 | Chthoniobacteraceae |
| 0 | 0 | 0 | 0 | 0.002568307 | Unclassified Alphaproteobacteria |
| 0 | 0 | 0 | 0.002355435 | 0 | Unclassified Phycisphaerales |
| 0 | 0.002220856 | 0 | 0 | 0 | Unclassified Ellin6529 |
| 0 | 0 | 0.00198415 | 0 | 0 | Unclassified WPS2 |
| 0 | 0 | 0 | 0 | 0.001983558 | Frankiaceae |
| 0.001773468 | 0 | 0 | 0 | 0 | Unclassified FW68 |
|  |  |  |  |  |  |
